# The Causes and Treatment of Habitual Constipation in Infancy

**Published:** 1898-08

**Authors:** 


### THE CAUSES AND TREATMENT OF HABITUAL CONSTIPATION IN INFANCY.

Dr. THOMAS S. SOUTHWORTH read a paper with this title. He said that from being regarded as a disease *per se*, amenable only to drugs, constipation had come to be looked upon as due to various functional disturbances of the organism. Much had been written from a theoretical standpoint regarding the peculiar anatomical conditions found in the sigmoid flexure, but his own observation on this point had led him to the opinion that their bearing upon the occurrence of constipation had been greatly exaggerated. Among the prominent causes of infantile constipation are deficient muscular power, disturbed peristalis, and altered consistency of the fecal masses. To these must be added the absence of voluntary effort in the infant. The speaker said that constipation in most fairly nourished infants yielded to a simple treatment which was largely dietetic. The fecal masses themselves should be inspected, dissolved and broken up by the physician, and in some cases even subject to chemical analysis. We should have more extensive analyses of the healthy, normal stools in the different periods of infancy so as to establish the variations within the limits of health. It had been shown that the milk of the nursing mother could be materially modified. The percentage of fat and the total quantity of the breast milk are the chief factors to be considered in connection with the subject of constipation. Too high a proteid percentage apparently produces looseness of the bowel and colic. The quantity of the mammary secretion could be increased by giving the mother more fluid, such as cow's milk, cocoa, thin gruels made from cornmeal and well-cooked flour. The extracts of malt increase the quantity of fat. Regurgitation by the infant of small quantities of milk after nursing usually indicates that the fat percentage has been increased too far. If the constipation be coincident with stationary weight, supplementary feedings are indicated. The stools would be found made up of small, firm scybalæ which, when broken up, are found to contain no curds, and seem to be well digested. A constipated child may show a fair gain in weight. Good results sometimes follow the addition of cream to the dietary given before each nursing, when the stools are dry and hard. Regulation of the mother's bowels should be undertaken, and occasionally assists in remedying infantile constipation. The commonest errors in diet leading to constipation are the giving of insufficient fat or proteid, or an excess of proteid. This insufficiency may depend upon excessive or insufficient dilution with water. Many children who thrive to all appearances on commercial condensed milk are constipated in spite of the large quantity of cane sugar present, because, as usually diluted, the fat and proteids are very low and the unabsorbed residue very small. The deficiency in the proteids results in a poor development of the muscles of the abdominal wall and of the intestine. To increase the amount of condensed milk is to increase proportionately the amount of cane sugar, which is not always advisable. The alternative is to change the food or to add a teaspoonful of cream for each teaspoonful of condensed milk. The same difficulty might arise where plain milk was given much diluted with water, and might be remedied by increasing the quantity of milk by adding cream or by the use of "top milk." The addition of both fats and proteids proves the most serviceable in the larger number of cases. One part of condensed milk represents only 2½ parts of ordinary milk. An error met with very commonly in artificial feeding is that of giving plain milk too diluted. If dyspepsia does not ensue, there are usually colic and constipation, the stools being hard, and when broken up, showing undigested casein. The proper dilution of the milk and the addition of cream will usually remedy the constipation. The use of well-cooked oatmeal gruel or jelly may sometimes be of service as a diluent for the milk. Certain non-alcoholic preparations of malt may occasionally be beneficial. The juice of half an orange may sometimes be given, twice a day, in the intervals of feeding, although it sometimes gives rise to troublesome urticaria.

Two special types of constipation remain to be considered. The first of these is the rhachitic, in which the diet must be regulated and the starchy elements reduced; the second, that form of chronic intestinal indigestion characterized by larger, light colored stools of the consistency of putty. The influence of habit in securing regularity of evacuation from the bowel has long been recognized, but is often not sufficiently appreciated. It has been found that if very young infants are placed over a warm chamber at regular intervals after feeding they will very quickly be induced to have regular evacuations. It is important that children old enough to sit at stool should be provided with a support for the feet, otherwise the abdominal muscles can not be properly brought into play. Abdominal massage would be found peculiarly useful in training the bowel to act at definite periods. The child should be laid on the back and the warmed hand introduced from below upwards underneath some light covering. The tips of the fingers should then be carried from the ileo-cecal region in small circles up to the transverse colon, then across and down along the descending colon to the region of the cecum, and then the process should be repeated, beginning at the same point as before. If the fingers are warm and the pressure is very light the child is not apt to cry. Five or ten minutes of such massage once or twice a day would usually be sufficient. No lubricant should be used, as it is desirable that the tissues underneath should be moved. At the conclusion of the seance, the child should be placed upon the chamber. When there is crying with defecation, and sometimes in its absence, anal fissures should be sought for.

Dr. SOUTHWORTH said that in his experience it was exceedingly rare to find a child who is artificially fed whose constipation cannot be remedied by an intelligent modification of the diet. At the beginning of the treatment the intestinal tract should be gently but thoroughly evacuated. For this purpose he preferred calomel in divided doses. It might be necessary at first to make daily use of mild laxatives during a gradual increase of certain elements in the previous diet, or the addition of new substances; but the laxatives should be reduced as soon as possible. Tablets of rhubarb and soda, of each 1½ grains, made up with oil of peppermint, may be dissolved and given two or three times a day, especially in the cases dependent upon disturbed peristalsis. It some cases the fluid extract of cascara sugrada, in doses of one to four minims daily, would answer well. The fluid preparations of malt were also efficient laxatives, either alone or combined with cascara. Cod-liver oil is a peculiarly serviceable remedy in those cases dependent upon poor nutrition, in which the addition of fat is indicated. In rather older children where a more decided action is necessary, more active drugs might be required. For comparatively short periods enemata may be employed with advantage, but they are extremely liable to abuse. When used occasionally they may be large, but when used daily, the smallest quantity that would secure an evacuation should be employed. Experience seems to show that cold injections excite more energetic contractions of the bowel than warm ones. Saline solution is less irritating than plain water. One teaspoonful of glycerine to a tablespoonful of water would be found an excellent means of securing a proper evacuation. Gluten or glycerine suppositories are useful, but medicated suppositories, when necessary, should be made of cocoa-butter, and the proportion of ingredients controlled by the physician's prescription; they should not be made up with glycerine.

Dr. A. JACOBI said that the paper was a good digest of what was known regarding the relation of infant feeding to infant digestion. The author's conclusions as far as modifications to the diet was concerned, were also rational, and, he believed, agreed with what most practitioners had found to be true. According to his own experience, a high proteid percentage did not give rise to diarrhea unless it had before excited constipation. Constipation of almost any kind, and particularly this variety, shows a large amount of cheese in hard, bullet-like stools. The truth of this statement could be easily determined by experience. A diet of proteids could, therefore, only relieve constipation by giving rise to diarrhea.

Regarding the action of cod-liver oil, he would say that if cod-liver oil acted in infants only as a food it could be replaced by other fats, such as cream. We knew, however, that its effect was different and more beneficial than that of any other fat. He believed that chemistry would eventually show that cod-liver oil contains substances comparable to what are now termed "internal secretions"—in other words, it was not simply a nutrient, but a specific remedy in a great many cases. He saw no reason why the intestine should not be washed just as regularly as the external integument. An enema need not be retained for any length of time; it should be given only to secure an evacuation of the bowel. He saw no reason why enemata should not be given for weeks or months or years; he certainly preferred them for securing an evacuation to the use of medicine given by the mouth. The addition of glycerine and soap and other substances to enemata made them irritating, which was an entirely different matter.

Regarding the anatomical peculiarities found in the intestine of the infant, Dr. Jacobi said that this important subject had been dismissed very summarily in the paper in a few words. The title of the paper included "the causes" of infantile constipation. If he had to classify the causes of such constipation, he would state that it is caused either by the condition of the intestine or the condition of the abdominal wall or that of the contents of the bowel. He was not one of those who believed that the bowels had little to do with the development of constipation. He had long ago called attention to the existence of a congenital constipation—a topic which did not yet appear in all the text-books. In the intestine of the fetus and of the infant there would be found an anatomical condition which gave rise to constipation of greater or less duration. This usually lasted only a year or two, but might last a number of years, or even a lifetime. The colon of the fetus and of the newly-born infant is about three times as long as the body of the infant; the colon of the adult is about twice as long as the body of the adult. On the other hand, the ascending colon and the transverse colon of the infant are very short; thus the excess of that colon must be covered by the length of the descending colon. Any one could convince himself of this by opening the abdominal cavity and determining by actual inspection that this was the condition present. Instead of one sigmoid flexure, as in the adult, there is a very long one, and sometimes two or three sigmoid flexures, one covering the other. Sometimes this flexure would be so greatly developed as to be found in the right side. More than forty years ago a French surgeon had insisted upon performing the operation for artificial anus in the young infant on the right side instead of the left, because of this condition. The length of the colon is the cause of the dessication of the feces. In the newly-born infant the colon is also quite narrow. These conditions give rise to a discontinuation of the downward progress of the feces, and hence the dryness and the constipation. He had described a case in which he had felt compelled to make an artificial anus in a newly-born infant. There was no passage of meconium, and the child was vomiting. The operation was done and the child died of peritonitis. The autopsy showed that the diagnosis was erroneous. There were three sigmoid flexures, one covering the other, and the feces of the upper part compressing the inner part.

The treatment of these cases of congenital constipation on general principles would prove disappointing; one must not expect a cure until the normal development of the colon had been completed. This form of constipation requires the daily use of enemata. It would save much medication and trouble, and probably much ill health to the baby. The anatomical development of the colon often did not reach its full development until the sixth or seventh year, and consequently this form of constipation often remains until that age.

When a baby, nursed at the breast with breast-milk, of normal appearance and quantity, is constipated from the very beginning, there must be a congenital condition giving rise to the constipation. Daily enemata constitute the appropriate treatment of this condition. Rhachitic babies are usually quite rotund in appearance, though somewhat more pale and flabby than usual. When two or three months old these children usually begin to suffer from constipation—indeed, constipation is often the first sign of rhachitis. The differential diagnosis between rhachitic constipation and anatomical or congenital constipation is, therefore, easily made.

The intestine may be influenced at a very early period in such a way as to give rise to subsequent constipation. Sometimes the intestine would be found a little constricted—indeed, certain portions might be said to be normally narrowed. Such conditions might, of course, very easily give rise to constipation. Another factor was ill-development of the muscular coat in portions of the intestine. This is the kind of constipation that may be helped by injections or massage. But there is another local impediment of great importance, not in the very young infant, but in late infancy and early childhood—i.e., peritonitis. When diarrhea lasts long in young infants it would never be confined to the mucous or submucous tissue, but it would penetrate to the serous membrane and cause peritonitis. Such localized peritonitis will, in time, lead to habitual constipation.

DR. FLOYD M. CRANDALL said that constipation was a subject in which it was comparatively easy to talk wisely, but very difficult to act wisely. Generalizations were easily made, but in the individual case it would be found very difficult to determine the cause or causes at work. There are two general reasons for failure in the treatment of constipation. The first one was that chronic constipation is a persistent condition and hence could not be cured by spasmodic and irregular treatment. The treatment must be carefully planned and systematically carried out. The second cause of failure was the complexity of the etiology of constipation. There were comparatively few cases which were not the result of a number of causes, and hence little aid could be expected from the use of one remedy or one measure. Undoubtedly the addition of fat to the food often relieves constipation, but there were many other things which usually require attention. He recalled a case of long-standing constipation in which a complete cure had been effected by the use of cold water between meals, but an attempt to follow out this treatment in any large number of cases, he said, would only result in disappointment and failure. The increase of the fat in the food would often lead to disturbance of the stomach. The successful treatment of constipation, therefore, consists in the use of a number of remedies and measures, and carrying them out according to a recognized plan.

DR. LEROY M. YALE said that he felt very much like the last speaker regarding the treatment of the particular case. He did not feel that high proteids generally caused diarrhea, unless they had first set up intestinal catarrh; on the contrary, a high proteid percentage was more apt to lead to constipation. In a general way it might be said that sugar is laxative, but this statement would really apply only to the coarse forms of sweets. Molasses, undoubtedly, is laxative, but refined sugar is not very commonly so. The sugar often causes fermentation, over-distension of the intestine, and constipation. As a rule, the physician could not show a distinct connection between the tendency of the child to become constipated and the constipation of the mother, but it was often due to the fact that it was difficult to get the children to take certain articles of diet. Different members of the same family would show remarkable differences in the effect of the same articles of diet. He agreed with Dr. Jacobi about the enema; he had lost his fear of it. It was true that the enema was often used needleesly, and that the mere insertion of the nozzle of the syringe would give a sufficient hint to secure the desired result. A matter of some importance is the proper position for defecation. The child is often put on a seat much too wide for it, and the flabby nates are crowded together and the evacuation interfered with. He knew of one case in which a child would not have a movement on the commode, but would get up and run to some other part of the room and there promptly have a natural stool. Another cause of constipation in children was the hurrying away to school in the morning, without taking time to go to the closet.

DR. SOUTHWORTH, in closing the discussion, said that he had desired to emphasize the importance of training the bowel of the infant from birth, to regularity, the adaptation of the food to the individual child, and the giving of as little medicine for constipation as possible. He had made the statement that he thought the question of anatomical or congenital constipation had been over-estimated because he had found many practitioners who seemed to think that this was the chief cause of constipation in infancy. Personally, he believed that constipation resulted from this congenital condition only in a comparatively small percentage of cases. It was only by the study of the individual case, and the use of different measures, that success would be achieved in the treatment of constipation.—*Pediatrics*.

